# In vitro co-culture studies and the crucial role of fibroblast-immune cell crosstalk in IPF pathogenesis

**DOI:** 10.1186/s12931-023-02608-x

**Published:** 2023-11-27

**Authors:** Fama Thiam, Sakshi Phogat, Filsan Ahmed Abokor, Emmanuel Twumasi Osei

**Affiliations:** 1 Department of Biology, University of British Columbia, 3187 University Way, ASC366, Kelowna, BC V1V1V7 Canada; 2 Centre for Heart Lung Innovation, St. Paul’s Hospital, Vancouver, BC Canada

**Keywords:** Pulmonary fibroblasts, Pulmonary immune cells, Idiopathic pulmonary fibrosis, Multicellular crosstalk, Co-culture models, Extracellular matrix, Fibrosis

## Abstract

IPF is a fatal lung disease characterized by intensive remodeling of lung tissue leading to respiratory failure. The remodeling in IPF lungs is largely characterized by uncontrolled fibrosis. Fibroblasts and their contractile phenotype the myofibroblast are the main cell types responsible for typical wound healing responses, however in IPF, these responses are aberrant and result in the overactivation of fibroblasts which contributes to the inelasticity of the lung leading to a decrease in lung function. The specific mechanisms behind IPF pathogenesis have been elusive, but recently the innate and adaptive immunity have been implicated in the fibrotic processes of the disease. In connection with this, several in vitro co-culture models have been used to investigate the specific interactions occurring between fibroblasts and immune cells and how this contributes to the pathobiology of IPF. In this review, we discuss the in vitro models that have been used to examine the abnormal interactions between fibroblasts and cells of the innate and adaptive immune system, and how these contribute to the fibrotic processes in the lungs of IPF patients.

## Introduction

Idiopathic pulmonary fibrosis (IPF) is a progressive, debilitating lung disease that has risen dramatically in incidence, with a 78% increase between the years 2000 and 2012 [1–4]. Currently IPF affects 0.33–4.51 per 10,000 persons globally [1–4]. IPF’s exact etiology remains elusive but has been associated with several risk factors including environmental (e.g., exposure to smoke, steel, brass, agriculture) and intrinsic (genetics, age, sex) factors [3]. IPF is a highly heterogeneous illness that results in an environment of chronic lung inflammation and fibrosis. The excessive fibrosis of the lung tissue leads to subsequent respiratory failure with a median survival of 3 years after initial diagnosis [5, 6]. There are currently two pharmaceutical agents approved by the Food and Drug Administration (FDA) for the treatment of IPF which are Nintedanib and Pirfenidone, that slow the decline in lung function and deterioration [7–9]. Although these treatments have been successful at slowing the progression of IPF, the disease remains incurable. Hence, there is an urgent need for research to determine (novel) underlying fundamental mechanisms of the disease to ultimately provide unique therapeutic targets that may lead to a cure.

The pathologic mechanisms that underline IPF have been shown to involve an exaggerated and abnormal response to wound healing and tissue repair [4, 5, 10]. Fibroblasts are crucial effector cells during normal repair and wound healing responses in healthy lung tissue [11–13]. As part of this process, fibroblasts proliferate and migrate to the site of injury where they are subsequently activated and differentiate into the highly synthetic and contractile myofibroblast phenotype [3, 12]. This activation is associated with (myo)fibroblast production of extracellular matrix (ECM) proteins (e.g., collagen) that support other cell types and restore damaged tissue during the repair processes [11, 14]. However, in IPF, fibroblasts are overactivated by the increased release of fibrogenic mediators from the pulmonary epithelium (e.g., transforming growth factor-β1 (TGF- β1), platelet derived growth factor (PDGF), connective tissue growth factor (CTGF), etc.) following lung injury [15, 16]. There is also the release of chemokines and cytokines from the damaged pulmonary epithelium and exaggerated immune response, that leads to the recruitment and activation of innate immune cells such as neutrophils and macrophages, that adds to the increased concentration of mediators to further activate (myo)fibroblasts [17, 18]. These innate immune cells in conjunction with the damaged epithelium then further activate the adaptive immune system (e.g., B cells and T cells) [18–20]. In various studies, it has been shown that multicellular interactions between the activated innate and adaptive immune cells and lung fibroblasts are crucial for the pathologic mechanisms of IPF. These interactions which occur through the production and release of fibrogenic mediators, impact lung fibroblast phenotype and function in the development of the fibrotic lesions that characterize IPF [18].

To assess and understand immune-fibroblast crosstalk in IPF, different in vitro models have been established. These diverse in vitro models that range from 3-dimensional (3D) hydrogel transwell co-cultures to decellularized lung scaffolds, incorporate immune cells and fibroblasts derived from IPF patients and healthy control individuals as well as continuous lung cell-lines and mimic the spatial configuration of cells in the in vivo environment to allow for the study of abnormal cellular communication [21, 22]. These representative (3D) in vitro models have allowed for the assessment of the specific processes involved in immune cell-fibroblast communication and how this contributes to IPF pathogenesis to help identify potential (novel) therapeutic targets.

In this review, we will summarize different studies that have used 2D and 3D in vitro models to examine the mediators and mechanisms involved in the crosstalk between pulmonary fibroblasts and immune cells in the pathogenesis of IPF. We will assess these studies in the context of the fibrotic mechanisms of IPF (including the production of ECM proteins, proliferation, migration, and apoptosis of lung fibroblasts) as well as how this interaction may lead to the production of inflammatory mediators that contribute to lung tissue remodeling in IPF. We then place these in the context of (future) therapeutic studies in IPF.

## Main text

### In vitro models used to examine fibroblast-immune crosstalk in IPF

To examine the role of fibroblast-immune cell interactions in IPF, various in vitro models have been utilized to mimic the microenvironment of the lung [22]. The simplest model used includes conditioned media-exposure experiments performed after culturing cells as traditional 2-dimensional (2D) monolayers before exposing one cell type (e.g., lung immune cells) to media harvested from the other cell type (e.g., lung fibroblasts) and vice versa [23]. This enables the investigation of the paracrine effects of mediators released from one cell type on the other exposed cell-type [24]. Other common models used are the in vitro co-cultures which have different variations (e.g., direct or transwell variations). The direct co-culture model involves culturing immune cells with lung fibroblasts directly in contact together in a tissue culture plate. In transwell co-cultures, lung immune cells are cultured in transwell inserts and lung fibroblasts grown in culture wells. Inserts with immune cells are then placed in wells with fibroblasts to establish transwell co-cultures [24, 25]. Additionally, 3D hydrogels established with natural (e.g., collagen I, gelatin) and artificial (e.g., polyethylene glycol, alginate) polymers have been used due to their ability to hold cells in a 3D spatial orientation and the ability to vary their matrix stiffness to mimic the in vivo tissue environment of the IPF lung more closely [26–28]. Here, fibroblasts and immune cells are either embedded together in 3D hydrogels or fibroblasts are embedded in hydrogels with immune cells seeded on top [28]. Co-culture models have been ideal for studying cell communication through the release of cellular mediators in the in vitro environment. However, these models are either established with plastic transwell inserts or reductionist natural ECM or artificial hydrogels. Hence, techniques have been developed that enables the decellularization of parts of the lung in vitro, which then serves as 3D scaffolds on which cells are cultured to assess their interactions in the natural pulmonary environment [29]. Further, lung tissue can also be used to generate thin slices about 100–500 μm thick, termed precision cut lung slices (PCLS) which is also used to study respiratory cell interactions in IPF [30]. PCLS are beneficial in vitro models because they can remain metabolically active while keeping the authentic structural integrity of the lung [30]. Ultimately, there is an abundance of in vitro models that can be employed and adapted to assess lung immune-fibroblast interactions in the pathobiology of IPF (see our reviews [25, 31, 32]. This review will integrate studies using these in vitro systems with specific emphasis on co-culture models to investigate the mechanisms and mediators behind the communication between fibroblasts and immune cells and how this contributes to the chronic lung tissue remodeling seen in IPF.

### Fibroblast-immune cell crosstalk contributes to fibrotic mechanisms in IPF

IPF is characterized by chronic remodeling of the lung tissue that involves excessive fibrosis which results in the destruction of the lung parenchyma, subsequently disrupting gas exchange and concluding in respiratory failure [6, 33, 34]. Fibrosis in IPF entails an established milieu of fibroblast derived ECM protein deposition with increased growth factor and cytokine release due to recurrent epithelial injury [18, 35]. The signaling mediators derived from the epithelium and other lung cells result in the overproduction and degradation of ECM proteins from fibroblasts, contributing to increased lung mechanical stiffness [6, 35, 36]. In addition to epithelial cells, immune cells such as macrophages, mast cells, B and T cells have also been shown to communicate with fibroblasts to contribute to increased synthesis and degradation of ECM proteins, which is important for aberrant matrix turnover and deposition in IPF [19, 37].

In line with the role of defective fibroblast-immune cell interactions in aberrant matrix turnover [38, 39], Bagher and colleagues, directly cocultured the LAD2 mast cell-line with either control or IPF-derived primary human lung fibroblasts (PHLFs) or HFL-1 human lung fibroblast cell-lines in culture plates or on decellularized lung scaffolds. It was found that IPF-derived PHLFs released significantly more hepatocyte growth factor (HGF) compared to control-derived PHLFs after co-culture with mast cells [38]. Further, it was found that α-smooth muscle actin (α-SMA) was upregulated in lung fibroblasts stimulated with TGF-β after co-culture [38]. HGF is a pleiotropic growth factor that contributes to fibrotic mechanisms by preventing apoptosis of structural cells and thus contributing to their abnormal activation and aggregation during defective injury repair [39, 40]. In corroboration with this, another study also found that mast cell-fibroblast interactions contribute to increased ECM proteins in IPF [41]. Here, Wygrecka et al., isolated IPF-derived primary lung mast cells and fibroblasts. The cells were then directly cocultured together which caused an increase in fibroblast synthesis of fibronectin and collagen I, that was found to be largely due to the release of the enzyme tryptase (a serine protease) by mast cells [41]. Hence in summary, coculture studies show that fibroblast-mast cell interactions increase the release of enzymes (e.g., tryptase) by mast cells as well as growth factors (e.g., HGF), and ECM proteins (e.g., α-SMA, fibronectin and collagen I) from fibroblasts which may directly contribute to fibrosis of lung tissue in IPF.

Further, Novak et al., isolated IPF and control-derived primary alveolar macrophages (AMs) and lung fibroblasts. Various combinations of CM experiments, direct co-cultures in tissue culture plates, transwell and 3D collagen hydrogel cocultures were then set up to assess the interaction between different combinations of IPF- and control- derived lung fibroblasts and AMs [42]. After experiments, it was found that while coculturing control-derived AMs with control-derived fibroblasts led to a reduction in fibroblast-α-SMA expression, direct cocultures of control-derived AMs with IPF-derived fibroblasts resulted in increased fibronectin, collagen I and III as well as α-SMA gene expression pointing to potential myofibroblast differentiation [42]. Lastly, it was found that IPF-derived fibroblasts expressed more collagen I and III when cocultured with IPF-derived AMs compared to control-derived AMs cocultured with IPF-derived fibroblasts [42]. In connection with this, Qu et al., also co-cultured IPF-derived primary lung myofibroblasts with the THP1 macrophage cell-line [43]. The fibroblasts were originally cultured on either stiff or soft polyacrylamide hydrogels coated in rat tail collagen I and treated with the Fas ligand (FasL), a type II transmembrane protein, to induce apoptosis [43]. After a phagocytosis assay, it was found that macrophages were able to clear fibroblasts cultured on soft matrix substrates (mimicking healthy lungs) than fibroblasts cultured on stiffer matrixes (mimicking IPF mechanical lung environment) due to FasL-dependent apoptosis [43]. In addition, it was discovered that the expression of death domain 1α (DD1α), a receptor responsible for the crosstalk between macrophages and cells undergoing apoptosis, is induced by the activation of the p53 transcription factor and was dependent on the expression of the gene, mouse double minute 4 (MDM4), a human mouse homolog, in soft matrix conditions [43]. Collectively, these studies showed that macrophage-fibroblast crosstalk contributes to IPF by causing the overproduction of ECM proteins such as collagen I, collagen III and fibronectin as well as causing a defective clearance of apoptotic cells which further advances the stiffening and scarring of the lung tissue.

In addition to macrophages, neutrophils are also important innate immune cells with elevated numbers in the lungs of IPF patients [44–46]. Although understanding the contribution of neutrophil-fibroblast crosstalk in IPF pathogenesis will add to our understanding of more crucial multicellular mechanisms, there is a lack of studies in this area due to the complexity of culturing neutrophils in vitro as they need to be freshly isolated from blood for every experiment and have a relatively short lifespan [47]. To account for this, neutrophil derivatives are applied to fibroblasts to examine their potential contributions to fibrosis in the context of IPF [48]. As an example, Gregory and colleagues exposed LL47 human lung fibroblasts to the enzyme, neutrophil elastase (NE) which is a protease that is able to break down proteins, and found an increased α-SMA production by fibroblasts with significant increases in pSMAD3, independent of TGF-β [48]. Further, NE exposed fibroblasts were then embedded in rat tail collagen hydrogels, where NE was found to enhance fibroblast contractility. Hence, this proves a potential neutrophil-fibroblast interaction in IPF that may cause fibrotic changes in fibroblasts (e.g., α-SMA increase) to advance IPF pathogenesis.

The adaptive immunity has also been identified as an important contributor to fibrotic mechanisms in IPF [18, 19, 49, 50]. In addition to innate immune cell-fibroblast interactions that result in increased fibrotic protein secretion, adaptive immune cell-fibroblast interactions seem to result in both the production and degradation of ECM proteins [51–53]. In line with this, Ali et al., isolated B cells from blood samples of healthy control individuals and IPF patients before stimulating them with (or without) either β-glucan or CpG [51]. β-glucan and CpG are microbial antigens which activate B cells via their pattern recognition receptors (PRRs) and mimic the microbial load in respiratory exacerbations in IPF [51]. Ali and colleagues then exposed IPF-derived fibroblasts to the CM from the stimulated B cells. CpG pre-stimulated B cell CM resulted in increased α-SMA, fibronectin and plasminogen activator inhibitor-1 (PAI1) in IPF-derived fibroblasts, whereas stimulation with β-glucan did not induce an activated phenotype in fibroblasts [51]. In connection with this, Selman et al., exposed lung fibroblasts to CM from IPF-derived primary T cells and found a significant increase in collagen synthesis from fibroblasts which they speculated could be due to the release of prostaglandin E (PGE) an eicosanoid mediator of inflammation and remodeling [52]. Further, Lacy et al., also isolated IPF- and control -derived T lymphocytes and exposed these to CD3/CD28 beads in media supplemented with IL-2 to activate them without adding antigen presenting cells [53]. In contrast to previous studies, Lacy et al., found that the direct co-culture of healthy T cells with control- or IPF-derived fibroblasts significantly reduced TGF-β induced myofibroblast differentiation, which was marked by decreased calponin and α-SMA [53]. Further, in a direct co-culture of IPF-derived T cells with both control- or IPF-derived fibroblasts, it was found that IPF-derived T cells reduced TGF-β-induced myofibroblast differentiation in both healthy and IPF-derived fibroblasts. Additionally, co-culture conditions did not increase control- or IPF-derived fibroblast expression of poly (ADP-ribose) polymerase (PARP), an apoptosis marker [53], suggesting there was no induction of cell death [53]. All the results obtained were also found under indirect co-culture conditions using Millicell hanging inserts, suggesting they are independent of cell-cell contact [53]. To summarize, direct and indirect transwell co-cultures and conditioned medium studies show that B cell-fibroblast crosstalk in IPF results in the upregulation of fibrotic proteins (e.g., α -SMA, fibronectin and PAI1), whereas T cell-fibroblast interactions in IPF are diverse, with increased fibrotic markers on one hand and a potential protective mechanism that cause decreased fibrotic changes on the other hand. The differences in T-cell-fibroblast crosstalk reported may be due to different experimental conditions and require further investigation to clarify roles and when these contribute to mechanisms of IPF [51–53].

**Fig. 1:**
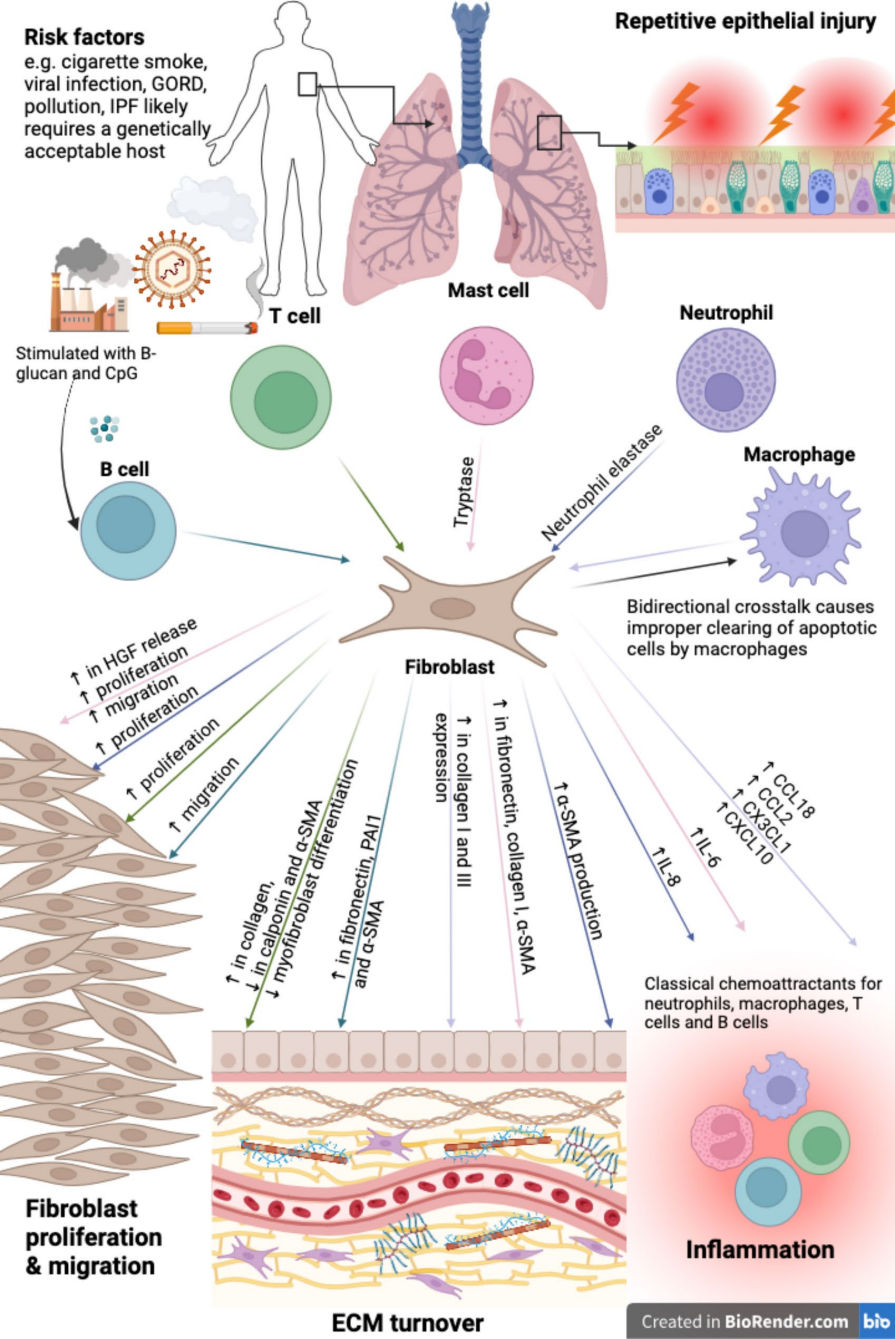
Mechanisms of immune cell-fibroblast interactions and how they contribute to the pathogenesis of idiopathic pulmonary fibrosis as determined by in vitro co-culture and conditioned medium model studies. Environmental toxins are inhaled into the lungs and cause repetitive injury to the epithelial layer in IPF pathogenesis. Recurrent epithelial injury causes the release of mediators that over-activate fibroblasts and attract immune cells. Fibroblasts interact with several innate immune cells resulting in various aspects of IPF pathobiology. Stimulated B cells interact with fibroblasts to increased migration in fibroblasts as well as to increase the synthesis of fibronectin, PAI1 and α-SMA. The crosstalk between T cells and fibroblasts result in increased proliferation of fibroblasts and increased collagen production. T cell-fibroblast interaction also causes a decrease in calponin and α-SMA and myofibroblast differentiation. Mast cell-fibroblast interactions are largely dependent on tryptase release, which alter fibroblast phenotype by increasing their proliferation and migration, as well as enabling the increased synthesis and release of HGF, fibronectin, collagen I, α-SMA and IL-6. Neutrophil elastase causes fibroblasts to release increasing amounts of IL-8 while a bidirectional crosstalk between fibroblasts and macrophages causes an increased expression of collagen I and III as well as the increased the release of CCL18, CCL2, CX3CL1 and CXCL10. Thus, crosstalk between various immune cells and fibroblasts contribute to IPF remodeling by triggering the overactivation of fibroblasts leading to their increased migration and proliferation which gives rise to fibroblastic foci, while also causing the overproduction and degradation of ECM proteins and contributing to the progressive accumulation of scar tissue, as well as causing the release of classical chemoattractants for immune cells (Figure created in Biorender.com)

Taken together, the studies presented in this section demonstrate the importance of the innate and adaptive immunity in regulating mechanisms of immune cell-fibroblast crosstalk that may drive fibrotic changes in IPF. Innate immune cells such as mast cells, macrophages and neutrophils all interact with fibroblasts through the release of growth factors and enzymes (e.g., HGF, tryptase, NE) that leads to increased ECM and structural proteins such as collagen, fibronectin, and α-SMA [38, 41–43, 48, 54]. The contribution of adaptive immunity to fibrosis through immune cell-fibroblast crosstalk seems to be more diverse, with interactions between activated B cells and fibroblasts resulting in upregulated ECM proteins while T cell-fibroblast crosstalk result in both increased collagen and decreased myofibroblast expression of α-SMA and calponin (Fig. 1). These studies are crucial to understanding the underlying mediators of the nuanced fibrotic processes that occur in IPF and will aid in potentially finding novel therapeutic targets for the disease.

### Fibroblast-immune cell crosstalk influences proliferation and migration and promotes apoptotic resistance in fibroblasts

The fibrotic mechanisms that characterise IPF have been shown to be impacted by the survival, proliferation, and migration of lung (myo)fibroblasts [55, 56]. In IPF, lung fibroblasts with abnormal (fibrotic) or myofibroblastic phenotypes often have prolonged survival rates due to processes that enable their resistance to apoptotic mechanisms [56, 57]. These mechanisms which include fibroblast-immune cell crosstalk, have also been shown to alter the proliferation and migration of defective lung fibroblasts/differentiated myofibroblasts which enables their accumulation in the IPF lung interstitium [58, 59].

To corroborate the role of lung fibroblast-immune cell crosstalk in mechanisms of fibroblast proliferation in IPF, Wygrecka et al., isolated IPF- and control -derived PHLFs and mast cells and co-cultured them directly [41]. The direct co-culture of IPF-derived human lung fibroblasts and mast cells rapidly induced increased proliferation of lung fibroblasts [41]. It was found that the high rate of fibroblast proliferation was largely due to an increase in the release of the enzyme tryptase by mast cells as the addition of the tryptase inhibitor, APC366, greatly attenuated the response [41]. The tryptase-mediated increase in lung fibroblast proliferation was also shown to be specifically mediated by the protease activated receptor-2 (PAR-2), which further induced the phosphorylation of protein kinase C-α (PKC-α), Raf-1, and p44/42. Hence, it was demonstrated that an interaction between the PKC-α/Raf-1/p44/42 pathway and PAR-2 receptor work together in advancing tryptase induced fibroblast proliferation due to mast cell regulation in IPF [41]. In connection with this, a study by Bagher and colleagues isolated peripheral blood derived human mast cells (PBdMC) and used LAD2 mast cell-lines in both direct co-cultures and CM experiments with the HFL-1 lung fibroblast cell-line [54]. When cells were co-cultured, PBdMC and LAD2 mast cells increased the migration of HFL-1 fibroblasts subjected to a scratch assay [54]. It was determined that the specific mast cell mediator responsible for increased migration and proliferation in HFL-1 fibroblasts was also the tryptase enzyme. The same group then established a similar model again, with the same PBdMC and LAD2 mast cells co-cultured with IPF-derived PHLFs in a subsequent study and found a similar result corroborating data from their first study [38]. Here, when IPF-derived PHLFs were exposed to mast cell mediators tryptase and chymase and scratch assays performed, it was shown that cells exposed to tryptase increased their migratory capacity while chymase had no effect on cell migration and even caused lower cell viability at high concentrations [38]. Interestingly, chymase also decreased the migratory capacity of lung fibroblasts when added to tryptase suggesting an antimigratory effect of chymase [38]. Taken together, through the use of co-culture models and CM exposure experiments, various studies have shown that mast cell-fibroblast interactions are crucial for increasing the proliferation and migration of fibroblasts through the activity of the enzyme tryptase [38, 41, 54]. This potentially adds to the increased accumulation of lung fibroblasts with defective fibrotic phenotypes in the IPF lung. However, the role of chymase in reduced fibroblast migration may provide a potential target for further studies in IPF disease therapy.

Due to the difficulty in culturing neutrophils in vitro, Gregory et al., used a neutrophil derivative to assess how human lung neutrophil-fibroblast crosstalk may influence defective fibroblast proliferation in IPF [48]. Here, the LL47 human lung fibroblast cell-line was stimulated with the enzyme NE which induced cell proliferation at modest concentrations accompanied by a complete loss of the insulin receptor substrate (IRS)-1, an intracellular mediator of phosphatidylinositol-3 kinase (PI3K) signalling [48] (a pathway implicated in cellular metabolism, proliferation, growth and survival) [60]. This presents a potential mechanism where human lung neutrophil-fibroblast interactions contribute to aberrant fibroblast proliferation in IPF via the PI3K pathway.

The interaction between adaptive immune cells such as T and B cells with fibroblasts have also been shown to influence fibroblast proliferation and migration in IPF. In a study by Chavez-Galan et al., human T cells enriched via negative selection from PBMCs isolated from the peripheral blood of healthy volunteers was exposed to CM collected from control- or IPF-derived primary lung fibroblasts [61]. IPF-derived fibroblast CM caused an increase in cell death of both CD4 + and CD8 + T cells [61]. Further, a human apoptosis array on both IPF- and control-derived fibroblast CM found a high concentration of secreted pro-apoptotic proteins including, pro-caspase 3, cytochrome C, hypoxia-inducible factor alpha (HIF-1α) and tumor necrosis factor receptor 1 (TNFR1) [61]. In the same study, cell migration was also assessed by stimulating T cells with CC chemokine ligand (CCL)2 (or monocyte chemoattractant protein 1 (MCP1)) and CCLl9 (both T cell chemokines), after healthy and IPF-derived fibroblast CM exposure [61]. Here, T cells previously exposed to IPF-derived fibroblast CM had decreased migration compared to cells previously exposed to control-derived CM after stimulation with CCL2 and CCL19 [61]. In connection with this,  study Selman and colleagues, exposed the fetal human lung fibroblast MRC-5 cell-line to primary human T cell-CM and found increased fibroblast proliferation rates. The authors suggested this could be due to secreted paracrine factors from T cells such as prostaglandin E [52]. Further to this, Ali et al., examined the role of B cell-fibroblast interactions in the context of IPF exacerbations by exposing IPF-derived primary fibroblasts to CM from B cells that were either unstimulated or stimulated with CpG or β-glucan [51]. Here, it was shown that activated B cell CM led to a significant increase in lung fibroblast migration compared to unstimulated B cell CM [51]. In summary, through mainly CM experiments, it has been shown that a bidirectional communication between the adaptive immunity represented by B and T cells and lung fibroblasts leads to decreased T-cell viability and migration on one hand and increased fibroblast migration and proliferation on the other hand. These different mechanisms point to the nuanced role of abnormal cellular crosstalk in complex diseases such as IPF. While abnormal activity of T-cells in some cases may be part of defective tissue remodeling, increased cell death of immune cells in a lung disease such as IPF may play a role in mechanisms that enable the persistence of abnormal (myo)fibroblasts in the lungs.

Taken together, the studies in this section suggest that lung immune cell-fibroblast interactions are crucial for aberrant rates of proliferation and migration of lung fibroblasts that add to remodeling mechanisms in IPF. The interaction between fibroblasts and innate immune cells lead to the release of mediators (e.g., NE for neutrophils, tryptase for MCs) that increase lung fibroblast proliferation and migration [38, 41, 48, 51, 54, 61]. Interactions between fibroblasts and cells of the lung’s adaptive immunity such as T and B cells through mediators such as PGE also contribute to lung fibroblast and immune cell migration and proliferation in IPF (Fig. 1). Comparatively, physical signals have been implicated in the attraction of macrophages to myofibroblasts. Pakshir and colleagues performed a co-culture of mouse primary bone marrow derived macrophages and lung fibroblasts and found that the force from the fibroblast contraction and fiber alignment was not sufficient to attract macrophages [62]. Chemoattraction of macrophages was dependent on the fibroblast’s deformation of the environment which than triggered attraction of macrophages via binding of α2β1 and stretch activated channels in the macrophages [62]. These findings are significant for the pathogenesis of IPF as lung fibroblasts are known to become aberrant, proliferate, migrate, and accumulate in the lung interstitium to form fibroblastic foci which are a major characteristic of the disease [63]. Utilising co-culture models to understand the mechanisms behind the formation of these foci and tissue remodeling in IPF such as immune-fibroblast interactions, present potential targets for novel therapies.

### Fibroblast-immune cell crosstalk contributes to inflammatory aspects of remodeling in IPF pathogenesis

In IPF, it is believed that repeated epithelial injury leads to chronic inflammation and the activation of various immune cells as well as fibrosis via the activation of (myo)fibroblasts [18, 64]. In different studies, crosstalk between fibroblasts and immune cells have been implicated in the production of inflammatory mediators that may contribute to the mechanisms of pulmonary remodeling in IPF.

To analyze the contribution of macrophage-fibroblast interactions in IPF, Prasse and colleagues isolated PHLFs and alveolar macrophages (AMs) from patients with IPF and healthy controls and used them to establish both direct and indirect co-cultures [65]. AMs used in co-cultures were either left unstimulated or stimulated with IL-4 and IL-10 which induced differentiation into M2 fibrosis-inducing macrophages [65, 66]. Direct human lung fibroblast-AM co-culture led to a significant increase in the release of the chemokine, CCL18 from AMs which was amplified when AMs had been differentiated into M2s via IL-4 and IL-10 stimulation [65]. Increased release of the chemokine, CCL18 is important for IPF inflammation as it is essential for recruiting adaptive immune cells such as B and T cells to perpetuate chronic inflammation [65, 67]. In connection with this, a 2023 study by Novak and colleagues exposed CM from healthy and IPF-derived AMs to control- and IPF-derived PHLFs [42]. Here, CM from healthy-derived AMs increased CCL2/MCP-1 in both IPF- and control-derived fibroblasts, however, IPF-derived AM-CM only increased CCL2 production in IPF derived-fibroblasts [42]. CCL2 is important for inflammatory mechanisms in IPF as it is a classical chemoattractant for T cells and monocytes [68]. Further to this, Qu et al. harvested CM from IPF-derived PHLFs cultured on polyacrylamide (PA) hydrogels with varying stiffnesses (soft to stiff, 5–20 kPa) and placed it in a transwell insert with the human THP1 macrophage cell-line cultured in suspension underneath [43]. Here, it was shown that CM from IPF-derived PHLFs cultured on softer matrices attracted more macrophages to migrate into the transwell insert than CM from PHLFs cultured on stiffer matrices [43]. This migration was found to be due to an increased concentration of CX3CL1 and CXCL10 proteins from lung fibroblasts which are considered classical chemoattractants for macrophages [43]. To summarise, through direct and indirect transwell co-cultures as well as CM experiments, it has been shown that the interaction between lung fibroblasts and macrophages lead to the release of cytokines and chemokines (e.g. CCL18, CCL2, CX3CL1 and CXCL10) that act as chemoattractants for immune cells that ultimately contribute to the inflammatory mechanisms involved in IPF chronic remodeling [42, 43, 65].

Mast cell-fibroblast interactions have also been implicated in inflammatory mechanisms that contribute to tissue remodeling in IPF [38]. Bagher et al., established co-cultures and found increased IL-6 release from PHLFs due to tryptase enzyme release from the LAD2 human mast cell-line [38]. IL-6 is a classical inflammatory cytokine that is known for the influx of neutrophils in the lungs [69]. In line with this, Amenomori and colleagues used a neutrophil derivative, human neutrophil peptide-1 (HNP-1), to stimulate normal human lung fibroblasts (NHLFs) which led to a trend in the increased release of the chemokine IL-8 from fibroblasts which is also a potent neutrophil chemoattractant [70]. Ultimately, through transwell co-cultures and neutrophil derivative experiments, potential mast cell and neutrophil – fibroblast interactions in the lungs via cytokines and chemokines (e.g., IL-6, IL-8) lead to the recruitment and activation of more immune cells (e.g., neutrophils) that perpetuate inflammatory mechanisms to add to pulmonary remodeling in IPF.

Taken together, these data demonstrate a distinct role for fibroblast-immune cell crosstalk in inflammatory mechanisms that may contribute to remodeling in the IPF lung. Here, lung fibroblasts may interact with macrophages to produce classical inflammatory mediators (e.g., CCL18, CCL2, CX3CL1 and CXCL1) that attract other immune cells to the site of injury in the lungs. The interaction between mast cells, neutrophils and fibroblasts lead to the release of IL-8 and IL-6 which are classical chemoattractants for neutrophils (Fig. 1) [69, 71]. These findings are crucial as increased neutrophils in the IPF lungs have been associated with IPF disease progession [72].

The interactions between immune cells and fibroblasts provide evidence for the presence of inflammatory mechanisms contributing to lung tissue remodeling in IPF [38, 42, 43, 65]. This notwithstanding, targeting inflammatory mechanisms in IPF with immunosuppressants have not yielded positive results in clinical trials [73–77]. In fact, as reported in the clinical trial, “Prednisone, Azathioprine, and N-Acetylcysteine: A Study That Evaluates Response in Idiopathic Pulmonary Fibrosis (PANTHER-IPF)”, a combination therapy of the named glucocorticoid and immunosuppressive agents, had to be discontinued as their administration was associated with increased death rate, hospitalizations, and adverse effects in IPF patients [74]. This suggests that further studies are needed to clarify the role of fibroblast-immune cell crosstalk-dependent inflammatory mechanisms in aberrant IPF tissue remodeling and how this may inform future therapeutic target studies and clinical trials.

### IPF therapeutic studies and lung immune cell-fibroblast crosstalk

Although there are two FDA approved drugs for the treatment of IPF, Pirfenidone and Nintedanib, the fact that the disease remains incurable, has led to further studies into the effect of the approved drugs on different IPF disease mechanisms such as aberrant immune cell-fibroblast interactions [33, 51, 78]. To assess this, Ali et al., treated B cells with Pirfenidone and Nintedanib before their activation via stimulation with CpG or β-glucan [51]. Here, it was shown that CM from Nintedanib treated, β-glucan and CpG-activated, B cells decreased the migration of lung fibroblasts whereas no results were observed when CM from Pirfenidone treated and activated B cells was placed on lung fibroblasts [51]. Further, it was determined that Nintedanib treated and activated B cell CM caused a decrease in the expression of fibronectin, PAI1 and α-SMA as well as vascular endothelial growth factor A (VEGFA) whereas Pirfenidone treated and activated B-cell-CM did not have such effects on lung fibroblasts [51]. Additionally, Nintedanib was found to decrease mTOR activation as well as decreasing Src and JNK phosphorylation where pirfenidone did not have any of these affects [51]. Further to this, Overed-Sayer, and colleagues also directly co-cultured cord blood-derived mast cells (CBMCs) with NHLFs on collagen coated plates [79]. It was found that the addition of Nintedanib abolished lung fibroblast-induced mast cell survival which was largely dependent on stem cell factor (SCF) [79]. SCF is a growth factor that is commonly overexpressed in IPF and functions in regulating proliferation and survival of mast cells [41]. Taken together, these studies show through CM and direct transwell co-culture experiments that, there is a clear role for Nintedanib-dependent regulation of B- and mast cell -mesenchymal crosstalk in the modulation of lung fibroblast migration and fibrotic gene expression as well mast cell survival, all of which are important (immune-fibroblast) pathologic mechanisms of IPF.

Although IPF is incurable, it has been shown that Nintedanib and Pirfenidone slow IPF progression through their antifibrotic effects which increases survival rates [80, 81]. In line with this, Behr et al., performed a survival analysis and found mortality rates to be decreased in individuals treated with Nintedanib and pirfenidone therapy compared to individuals not treated with antifibrotic therapy [80]. Further, Margaritopoulos and colleagues found that Pirfenidone specifically, had increased survival rates of approximately 30% compared to untreated IPF patients [81]. However, despite the advantages of the current treatments, IPF still remains incurable with mortality rates ranging from 0.5 to 12 per 100,000 of the population worldwide [82]. Hence, using unique co-culture models as presented in this review will enable the assessment of potentially novel mechanisms of action for the approved drugs in a bid to assist with patient specific precision medicine [83]. Further, the interaction of Pirfenidone and Nintedanib with the specific mechanisms of fibrosis discussed in this review, have a high potential to aid in future drug development studies for IPF. Parallel to this, there is also the potential to discover novel therapeutic targets and agents for IPF through these complex co-culture studies. Here, studies such as what was done by Lacy et al., [53] where T cells were able to inhibit myofibroblast differentiation, are critical to finding innovative mechanisms which can be further assessed for therapeutics.

### Future indications for in vitro models assessing IPF immune cell-fibroblast interactions

This review summarized various studies that established in vitro (transwell) co-culture and CM exposure models to assess the role of immune cell-lung fibroblast interactions in various mechanisms such as fibrosis, fibroblast migration and proliferation as well as inflammatory processes associated with the chronic remodeling of lung tissue in IPF (See Table 1). In addition to the in vitro co-culture models discussed here, other complex biomimetic or bioartificial models such as 3D bioprinted lung tissue models, microfluidic lung-on-a-chip systems, PCLS and 3D lung organoids are also being explored as (novel) in vitro systems to assess complex multicellular interactions in IPF [21, 84–86].These interactions transcend immune cell-fibroblast crosstalk and may involve communication with other pulmonary cell types such as the epithelium and endothelial cells [84–86]. Although most of the other models mentioned have also assessed crosstalk between two cells, (e.g., epithelial-fibroblast interaction [25, 31, 32, 87–89], they provide great potential to further assess multicellular interactions between more than 2 cell types (e.g., epithelial-fibroblast-immune-cell crosstalk). In line with this, recent digital spatial profiling studies performed on different regions of interest in IPF tissue compared to healthy control tissue revealed increased gene expression of ECM proteins (e.g., Tenascin C, fibrillar collagens (COL1A2)) in IPF fibroblastic foci. Through bioinformatic ligand-receptor interaction analysis it was shown that adjacent alveolar septae potentially signaled through mediators such as TGF-β1, bone morphogenetic protein 4 (BMP4), CCL2, CD24, HGF, secreted phosphoprotein 1 (SPP1) and the plasminogen activator, urokinase (PLAU), while immune infiltrates signaled through TGF-β1, high mobility group box 1 protein (HMGB1), CD24 and SPP1 to fibroblast foci. Further, an upregulation of CXCR4, the receptor of the cytokine CXCL12 (known to exclude T-cells in cancer) was linked to increased CXCL12 and a downregulation of NF-kappa-B inhibitor zeta (NFKBIZ) in fibroblast foci. Through gene editing experiments, it was shown that the downregulation of NFKBIZ led to reduced mRNA and protein expression of TGF-β1-induced IL-6 in alveolar epithelial cells. Hence, this study revealed potential interactions between immune infiltrates, diseased fibroblastic foci and alveolar septae in IPF that may downregulate inflammatory mechanisms (IL-6 activity) that inhibit fibrosis. As most of this data was observational, and added to the fact that clinical trials with the monoclonal antibody tocilizumab targeting the IL-6 pathway in IPF have increased the risk of non-infectious pulmonary complications [90], it is important to further assess the nuanced spatio-temporal mechanisms of crosstalk with immune-epithelial-fibroblast triculture systems that improves on the models described in this review to reveal novel therapeutic targets. Most 2D and 3D co-culture systems have been developed as proof-of concept models and have been used for low to medium through-put studies. The adaptation of these systems for high-throughput set-ups is an area of active research and most likely involves the combination of bioengineering, high-level automation, and artificial intelligence techniques/methods. An area of biomimetic model studies that is seeing improvements with regards to high-throughput techniques involves the development of multicellular spheroids or organoids which can be done via bioprinting techniques where a mixture of cells and Matrigel (basement membrane proteins) are loaded into a printer which accurately prints drops of cell-embedded Matrigel in which organoids form. The liquid overlay technique where cells are seeded on non-adhesive substrates for rapid aggregation is also efficient and reproducible for the formation of spheroids [91, 92]. The application of these methods would have significant benefits for therapeutic research in pulmonary diseases such as IPF.

**Table 1:** Summary of in vitro co-culture and conditioned medium model studies assessing immune-fibroblast interactions in the pathogenesis of idiopathic pulmonary fibrosis

In Vitro Models	Description	Mediators involved	Finding	Refs
Direct coculture 2D and 3D on plates or decellularized lung scaffolds and CM	LAD2 mast cell line directly cocultured with PHLF’s or HFL-1	Mast cell tryptase, Mast cell Chymase, Fibrogenic release of VEGF, HGF and IL-6	Upregulation of ECM proteins and growth factors that prevent apoptosis of endothelial and epithelial cells and contribute to the stiffening of the lung tissue. Increase in fibroblast migration. Contributes to inflammatory mechanisms by attracting neutrophils	[38]
Direct coculture	HLF’s isolated from control or IPF lungs cultured with primary human mast cells on tissue culture plates	Mast cell release of tryptase, Fibrogenic fibronectin and collagen I release	Increased release of fibrogenic ECM proteins contributes to the fibrosis of lung tissue. Increased proliferation of fibroblasts	[41]
Direct co-culture and indirect transwell co-culture Direct co-culture in 3D hydrogels CM	IPF and control-derived primary alveolar macrophages and lung fibroblasts	Macrophage release of CCL2 Fibrogenic release of fibronectin collagen I &III	Contributes to inflammatory mechanisms by attracting immune cells. Increase ECM proteins, potential myofibroblast differentiation.	[42]
CM	Primary IPF fibroblasts with THP-1 macrophages, treated with FasL to induce apoptosis	MDM4 gene expression regulates P53 activation of DD1α receptor on macrophages. Macrophage release of CX3CL1 and CXCL10	Improper clearing of apoptotic cells on stiff matrices. Chemokines and cytokines attract immune cells.	[43]
Neutrophil derivative Exposure model	Lung fibroblasts exposed to Neutrophil Elastase (NE).	Neutrophil elastase stimulate fibrogenic release of pSMAD3 & α-SMA. Loss of the insulin receptor substrate (IRS)-1, an intracellular mediator of phosphatidylinositol-3 kinase (PI3K) signalling	Increase in fibrogenic phenotype of fibroblasts. Increase in fibroblast proliferation	[48]
CM	IPF-derived fibroblasts exposed to CM from B cell stimulated with bacterial antigens (β-glucan & CpG)	Fibrogenic increase in the expression of of PAI1, α -SMA and fibronectin	Upregulation of ECM proteins, increase in fibroblast migration. Nintedanib treated B-cell CM decreased migration of lung fibroblasts and fibrogenic phenotype	[51]
CM	Lung fibroblasts exposed to CM from IPF-derived primary T cells	Authors suggested this could be due to factors such as PGE2	Increase in fibroblast collagen production and proliferation	[52]
Direct co-culture Indirect transwell coculture	IPF derived T cells co-cultured with control and IPF-derived fibroblasts	Decreased calponin and α -SMA	T cells decrease myofibroblast differentiation induced by TGF-β.	[53]
Direct co-culture and CM	PBdMC and LAD2 mast cells with HFL-1 lung fibroblasts	Mast Cell Tryptase	Increased fibroblast migration and proliferation	[54]
CM	Healthy human primary T cells exposed to CM from IPF-derived primary lung fibroblasts	High concentration of pro-apoptotic proteins in fibroblast CM including Pro-caspase 3, cytochrome C, HIF-1 α and TNFR1	Increase in CD4 + and CD8 + cell death, decrease in T cell migration after chemokine exposure.	[61]
Direct and indirect co-cultures	IPF and control-derived primary human lung fibroblasts and alveolar macrophages co-cultures	Alveolar Macrophage release of the chemokine CCL18	Potentiates inflammation by attracting adaptive immune cells	[65]
Neutrophil derivative Exposure model	HNP-1 exposed to normal human lung fibroblasts	Fibrogenic release of IL-8	Leads to the recruitment and activation of neutrophils	[70]
Direct co-culture	Co-culture of cord blood-derived mast cells (CBMCs) with normal human lung fibroblasts on collagen coated plates	Mast Cell Stem Cell Factor release	Addition of Nintedanib abolished lung fibroblast induced mast cell survival	[79]

It should be noted that in addition to their involvement multicellular interactions, defective immune cell phenotype and function have been implicated in IPF disease mechanisms and significantly associated with disease outcomes. In line with this, various immune cells such as monocytes, neutrophils, B cells and T cells have been shown to be elevated in the bronchoalveolar lavage fluid from IPF patients [18, 93]. In line with this, neutrophilia in IPF patients has been strongly associated with disease progression and early death [18]. Further, increased levels of blood monocytes have been associated with lower survival rates as opposed to increased resting memory T cells that have been linked with increased survival rates in IPF patients [94–96]. Additionally, macrophages have been found to accumulate in the lung parenchyma and produce the fibrotic mediator osteopontin that is implicated in remodeling and fibrosis of the lung tissue [97]. The current manuscript analyzed and assessed studies that have researched the involvement of these immune cells in defective multicellular interactions with lung fibroblasts in IPF. There are various reviews that address the individual relevance of the immune cells described here in the pathogenesis of IPF [44, 98, 99].

Further, this review analyzed studies assessing fungal and bacterial infection in relation to fibroblast-immune cell crosstalk in IPF, via stimulating B cells with CpG and β-glucan antigens. Although fungal and bacterial infections are an important aspect of disease, the presence of chronic viral infections have also been shown to be a significant risk factor and linked to poor disease outcomes in IPF [100–102]. However, not much work has been done assessing the role of viral stimulations in aberrant fibroblast-immune cell crosstalk in IPF. Hence, further work assessing this will shed light on an important aspect of the disease.

As detailed in this review, in vitro co-culture models have contributed to preclinical research in IPF and other lung diseases [25, 31, 32, 87–89, 103] over the years, however there are caveats associated with the use of these models. First in most of the 2D co-culture and simpler conditioned medium exposure studies, the experimental set-up does not mimic the spatial 3D configuration of tissues in the lung microenvironment, although these models improve upon traditional 2D monolayer single cell culture set-ups. In line with this, 2D co-culture and conditioned medium exposure models still do not overcome the limitations of altered cellular phenotype and function due to cell culture on stiff plastic plates or glass slides as opposed to 3D soft tissue [104]. These limitations are addressed in co-culture models where cells are embedded in or cultured on 3D hydrogels made from ECM proteins and their derivatives such as collagen-I or gelatin. We have reviewed the various variations of 3D co-cultures elsewhere [25, 32, 87, 105, 106]. In both 2D and 3D co-cultures an important drawback to consider is the need for careful optimization of cell culture media to enable increased cell viability of both cells. This drawback is potentially amplified when co-cultures are established with primary human lung cells as different immune cells and fibroblasts require different supplements and growth factors that may inhibit each other’s growth. Therefore, in preliminary experiments, it is crucial to determine the proportions of growth media from the different cells being co-cultured that can be mixed to achieve optimal growth of both cells. This step can be time and resource consuming. Further, in more complex 3D co-cultures such as those established with hydrogels, there needs to be careful consideration of cell isolation and sorting techniques that can allow downstream assays such as protein and RNA analysis to be completed. Again, most of the models described in this review are static co-culture systems which do not account for the mechanodynamic pulmonary environment due to air and blood flow. These are hopefully counteracted in more recent and complex models such as lung-on-chip systems. Ultimately, in vitro models are still reductionist systems that are not able to fully capture the complex physiology, architecture and all the multicellular environment in the human body. Advances in this field through more complex systems are, however, addressing these shortcomings. The data obtained from 2D, and 3D co-cultures, however, still offer insights into nuanced disease mechanisms that were hither-to unknown or understudied.

Complex biomimetic in vitro models may provide a more wholistic representation of the 3D orientation of lung architecture as opposed to co-culture models. Hence, taken together, complex bioartificial co-culture models present an enormous potential for the study of novel IPF disease mechanisms that will help identify future therapeutic targets needed to ultimately develop a cure for chronic remodeling in IPF.

## Conclusions

In conclusion, this review examined the mechanisms behind lung fibroblast-immune cell communication via in vitro (transwell) co-culture models and how they contribute to the various mechanisms of chronic remodeling in IPF. A common significant finding was that the interaction between lung fibroblasts and different lung immune cells in both the innate and adaptive immunity such as mast cells, neutrophils, macrophages, B cells and T cells result in an increase in fibroblast proliferation and migration as well as alterations in lung fibroblast activation and ECM protein deposition which are crucial mechanisms involved in excessive fibrosis and lung tissue remodeling in IPF. Specifically, immune cell-fibroblast crosstalk contributes to fibroproliferative mechanisms in IPF because of interactions through mediators, growth factors and enzymes such as HGF, PGE, tryptase and NE. Interestingly, immune cell-fibroblast interactions through mediators such as CCL18, CCL2, CX3CL1, CXCL1, IL-6 and IL-8 also contribute to inflammatory mechanisms that may add to tissue remodeling in IPF and need further investigation to ascertain the role of inflammatory mechanisms in IPF therapeutics. In line with this, through these studies, a protective role of T-cells on fibroblast activation and fibrotic phenotype has been uncovered that may serve as a basis for further (therapeutic) studies. These and other disease processes which have also been shown to be targeted as part of the mechanism of action for the antifibrotic drug Nintedanib, points to how crucial multicellular co-culture model studies are in understanding nuanced and complex disease mechanisms and eventually discovering a cure for IPF.

## Data Availability

NA.

## List of abbreviations

IPF: Idiopathic pulmonary fibrosis
FDA: Food and Drug Administration
ECM: extracellular matrix
TGF: β-transforming growth factor-β
PDGF: platelet derived growth factor
CTGF: connective tissue growth factor
3D − 3: dimensional
2D − 2: dimensional
µm: micrometer/microns
CM: Conditioned Medium
PCLS: precision cut lung slices
MC: Mast cells
PHLFs: primary human lung fibroblasts
NHLFs: normal human lung fibroblasts
LAD2: mast cell line
HFL: 1-human lung fibroblast cell-line
HGF: hepatocyte growth factor
α: SMA-α-smooth muscle actin
AMs: alveolar macrophages
THP1: macrophage cell-line
FasL: Fas ligand
DD1α: death domain 1α
MDM4: mouse double minute 4
LL47: human lung fibroblasts
NE: neutrophil elastase
pSMAD3: phosphoSMAD3
CpG: Oligodeoxynucleotides containing unmethylated CpG motifs
PRRs: pattern recognition receptors
PAI1: plasminogen activator inhibitor-1
PGE: prostaglandin E
CD: Cluster of Differentiation
IL: Interleukin
PARP: poly (ADP-ribose) polymerase
APC366: selective inhibitor of mast cell tryptase
PAR: 2-protease activated receptor-2
PKC: α-protein kinase C-α
Raf: 1-rapidly accelerated fibrosarcoma − 1
PBdMC: peripheral blood derived human mast cells
IRS: insulin receptor substrate
PI3K: phosphatidylinositol-3 kinase
PBMC: Peripheral Blood Mononuclear Cell
HIF: 1a-hypoxia-inducible factor alpha
TNFR1: tumor necrosis factor receptor 1
CCL: CC chemokine ligand
CXCL: CXC chemokine ligand
MCP1: monocyte chemoattractant protein 1
MRC: 5-fetal human lung fibroblast cell-line
M2: fibrosis-inducing macrophages
PA: polyacrylamide
HNP: 1-human neutrophil peptide-1
VEGFA: vascular endothelial growth factor A
CBMCs: cord blood-derived mast cells
SCF: stem cell factor
COL1A2: fibrillar collagen I alpha 2
BMP4: bone morphogenetic protein 4
SPP1: secreted phosphoprotein 1
PLAU: plasminogen activator, urokinase
HMGB1: high mobility group box 1 protein
NFKBIZ: NF-kappa-B inhibitor zeta
